# Impact of COVID-19 restrictions on motor coordination in Italian school-aged children

**DOI:** 10.3389/fspor.2026.1861717

**Published:** 2026-08-12

**Authors:** Valerio Giustino, Jessica Brusa, Roberta Cottone, Marianna Bellafiore, Federico Schena, Valentina Biino

**Affiliations:** 1 Sport and Exercise Sciences Research Unit, Department of Psychology, Educational Science and Human Movement, University of Palermo, Palermo, Italy; 2 Department of Human Sciences and Promotion of the Quality of Life, San Raffaele University, Rome, Italy; 3 Department of Neurosciences, Biomedicine and Movement Sciences, University of Verona, Verona, Italy

**Keywords:** gross motor coordination, motor competence, motor development, pandemic, physical abilities, physical fitness

## Abstract

**Introduction:**

The development of motor coordination (MC) is essential during childhood and is closely influenced by opportunities for physical activity (PA) and sports participation. The COVID-19 pandemic and related restrictions may have altered children's motor experiences and the related MC development. To what extent MC has changed in Italian school-aged children from before the pandemic until its end and beyond remains unclear. The aim of this study was to compare the level of MC in Italian school-aged children aged 8–10 years from three independent cohorts (i.e., pre-COVID-19, post-COVID-19, and follow-up COVID-19 restrictions).

**Methods:**

Overall, 449 school aged children aged 8–10 years were included from three independent cohorts (i.e., pre-COVID-19 *n* = 141; post-COVID-19 *n* = 139; follow-up COVID-19 restrictions *n* = 169). The Körperkoordinationstest für Kinder (KTK) was used to assess children's MC. The test consists of four subtests: walking backward (WB) on beams of decreasing widths; moving sideways (MS) on the floor for 20 s; two-legged jumping sideways (JS) over a beam for 15 s; one-legged hopping for height (HH) over an obstacle of increasing height. A Motor Quotient (MQ) was calculated from the sum of the four subtests' scores. The children's PA level was assessed using the Physical Activity Questionnaire for Children (PAQ-C), referring to the previous seven days. All assessments were conducted during physical education classes by the same researchers.

**Results:**

The three-way ANOVA revealed significant effects in the Time factor (i.e., pre-COVID-19; post-COVID-19; and follow-up COVID-19 restrictions) in all the subtests. We detected significant reductions from pre- to post- and from pre- to follow-up in all the subtests (except from pre- to post- for the WB subtest), as well as for the MQ score (*p* < 0.001). A significant effect in the Sex factor was also found, with females showing lower MC levels than males (*p* = 0.002). Regarding PA levels, significant reductions from pre- to follow-up (*p* = 0.006) and from post- to follow-up (*p* = 0.002) were observed, with males reporting higher PA levels than females (*p* = 0.005).

**Discussion:**

When comparing the pre-pandemic cohort with both the post-pandemic cohort and the follow-up cohort, we found higher MC performances. Hence, the COVID-19 restrictions were associated with decreased MC levels in Italian school-aged children aged 8–10 years.

## 1 Introduction

Motor coordination (MC) refers to the ability to move all parts of the body efficiently and fluidly to execute motor skills in time and space (1, 2). It can be categorized in general, specific, and inter-segmental reflecting the ability to perform activities of daily living, to manage sports gestures, and to control degrees of freedom of different body parts employed during movements, respectively (1–3). Furthermore, MC can be divided into gross, including actions that require large muscle groups (e.g., walking or jumping), and fine, including actions in which small muscle groups are involved (e.g., hand movements such as writing) (4, 5).

The golden age for MC development has been established between 5 and 6 years (5) with the greatest adaptability of MC skills between 7 and 12 years (6). In fact, it is well known that the development of MC during childhood is fundamental for everyday tasks and, moreover, it is a predictor of physical activity (PA) practice and also promotes participation and success in sports (6–8). It should be mentioned that, in the opposite way, the practice of PA or sports also improves MC, thus establishing an interrelationship between them (9–11). Based on the latter, as each sport has specific physical demands, different research groups demonstrated that sport participation can lead to early specialization, that is the practice of a sport than others can improve some sport-specific characteristics of MC (12, 13). Moreover, the more a sport it is practiced, the higher the level of MC acquired will be (14), although young who engage in multisport activities seems to perform better in MC than those specialized in a single sport (15).

Conversely, not practicing PA or sports, as well as not reaching the amount of PA recommended (16), leads to a poor or a not adequate level of MC as amply demonstrated previously (2, 17, 18). For these reasons, the COVID-19 pandemic and the associated restrictions on PA and sports might have led to changes in MC, reflecting the alternating phases of restrictions. This may be particularly noticeable in children belonging to those ages in which the development of MC can be sensitive and susceptible to various factors, including the practice of PA and sports. Indeed, in many countries worldwide, several restrictions were adopted, including the closure of sports facilities and the prohibition of access to public parks and gardens, to limit the spread of the virus during the pandemic (19, 20). In Italy, the state of emergency was declared on January 31, 2020, and ended on April 1, 2022. During this long period, several Decrees of the President of the Council of Ministers (DPCM) were issued. In detail, the main DPCMs that limited, regulated, or suspended PA and sports practice can be categorized as those of the first wave (March–May 2020) and the second wave (October 2020–December 2020). However, various restrictions (March 2021 and subsequent ordinances) continued to be issued throughout the country, depending on the risk zones.

Several authors demonstrated a reduction of physical fitness in children during COVID-19 lockdown (21–24). For example, a study used data from a sample of 24,500 Austrian primary school aged children showed that physical fitness features were significantly lower post-COVID-19 pandemic compared to pre-pandemic period (25). Similar findings were detected in a cohort of 125,893 German school-aged children aged 8–10 years in which lower physical fitness and coordination level were associated with COVID-19 pandemic (26). The existing literature suggests that Italian children have also shown a reduction in physical fitness and MC compared to the years prior to restrictions and this aspect can be associated with the reduction in the practice of PA and sports (27, 28). Nevertheless, how COVID-19 restrictions and the long-term effects of COVID-19 restrictions were associated with changes in Italian children's MC are not clear.

Hence, the aim of this study was to compare the level of MC in Italian school-aged children aged 8–10 years from three independent cohorts (i.e., pre-COVID-19, post-COVID-19, and follow-up COVID-19 restrictions). The hypothesis was that children's MC levels reflected the different phases related to pandemic restrictions. Specifically, immediately after pandemic restrictions (i.e., in 2022), children showed lower MC levels than before the pandemic (i.e., in 2019), and that in the long term (i.e., in 2025), MC levels increased, although these did not return to pre-pandemic levels.

## 2 Methods

### 2.1 Study design

This study is part of the project “Gross Motor Coordination in Italian children: a cross-sectional study comparing age-matched cohorts assessed in different years”. This project aims to establish the normative values of Gross Motor Coordination in Italian school-age boys and girls, and the association between MC and PA in order to enhance the role of PA and Physical Education (PE) in the school context from Northern and Southern Italy.

For this study we included Italian school-aged children between 8 and 10 years from three independent cohorts (i.e., in 2019, in 2022, and in 2025, for comparing data pre-COVID-19, post-COVID-19, and follow-up COVID-19 restrictions, respectively) belonging to a school in Northern Italy (i.e., Verona city) and a school in Southern Italy (i.e., Palermo city).

The multicentre study was approved by the Ethics Committees of the University of Verona (No. 2019-UNVRCLE-0298910) and University of Palermo (No. 8/2019), in accordance with the principles of the Declaration of Helsinki (29).

All children recruited for the study were eligible to participate with prior written informed consent provided by their parents or legal guardians.

### 2.2 Participants

The total sample of the three independent cohorts consisted of 449 children (*n* = 225 from Northern Italy and *n* = 224 from Southern Italy) of which *n* = 141 pre-COVID-19, *n* = 139 post-COVID-19, and *n* = 169 follow-up COVID-19 restrictions. The participants were in third, fourth, and fifth grade of primary school. The inclusion criterion was that school-age children were between 8 and 10 years old.

Data from the assessments of the first cohort (i.e., in 2019, that is pre-COVID-19) were drawn from our previous studies (30, 31) that investigated the influence of geographical area (Northern vs. Center vs. Southern Italy), living setting (rural vs. urban), age, sex, and body weight status on MC and PA levels.

Data from the assessments of the second cohort (i.e., in 2022, that is post-COVID-19) were drawn from our previous study (12) aimed at examining whether the participation in sports could influence the level of MC compared to peers who did not practice any sport, and at comparing MC in children who practiced different sports.

Data from the assessments of the third cohort (i.e., in 2025, that is follow-up COVID-19) were collected from a school in Northern Italy and a school in Southern Italy with the aim of investigating how the long-term effects of COVID-19 restrictions were associated with MC, with particular attention to age and sex factors.

### 2.3 Procedure

The anthropometric and MC assessments were conducted during PE classes, therefore during school hours. This allowed to collect data in the same time slot (from 8.00am to 13.00am). Furthermore, assessments were carried out in the same places (the gyms of the schools) and by the same researchers. The questionnaire used to measure the amount of PA practiced was completed by children at school, and during school hours, under the supervision of the same researchers.

The presence and collaboration of PE teachers was always ensured to instil confidence in children. The researcher-to-student ratio was 1:2.

#### 2.3.1 Anthropometric assessments

Anthropometric measurements were performed according to the standardized protocol described by Clarys et al. (32). Height was measured with a stadiometer to the nearest 0.5 cm. Weight was measured with an electronic scale to the nearest 0.1 kg. For anthropometric measurements, each participant wore minimal clothing and was barefoot. Then, body mass index (BMI) was calculated using height and weight [(weight (kg)/height^2^ (m^2^)] (33, 34).

#### 2.3.2 Motor coordination assessment

The Körperkoordinationstest für Kinder (KTK) was used to assess children's MC (35, 36). The KTK demonstrated well established reliability and validity (*r* = 0.97; WB = 0.80; JS = 0.95; MS = 0.85; HH = 0.96) (35, 36). The test consists of four subtests: (1) walking backward (WB) on beams of decreasing widths; (2) moving sideways (MS) on the floor for 20 s on plates; (3) two-legged jumping sideways (JS) for 15 s over a beam; (4) one-legged hopping for height (HH) over an obstacle of increasing height. A previous study, investigating the internal validity of the KTK, showed very high associations between scores of subtests and the total score of the KTK, although a short form does not seem to be fully satisfactory for assessing MC in children (37).

The KTK was administered following the standardized and validated procedure of the test developed by Kiphard and Schilling (35, 36).

Before test administration, a detailed verbal explanation was provided, accompanied by verbal encouragement from the researchers.

The WB subtest consisted of walking backward on three wooden beams, each 3 m long and 8 cm high, with decreasing widths (i.e., 6.0 cm, 4.5 cm, and 3.0 cm). After an initial familiarization phase involving walking both forward and backward on each beam, each participant performed three trials on each beam. The task consisted of performing up to eight consecutive steps on each beam without touching the floor. The score was calculated as the total number of steps performed correctly, with a maximum score of 72 (8 steps × 3 trials × 3 beams).

The MS subtest consisted of moving sideways as quickly as possible in 20 s on two wooden boards (25 cm × 25 cm × 5.7 cm). After an initial familiarization phase in which the test was tried, each participant performed two trials. The task consisted of standing with both feet on a wooden board with a second wooden board placed a few centimetres to one side of the first. Each participant moved sideways from the first board to the second, then lifted the free board with their hands, positioning it next to and on the other side of the one they were standing on, and then stepped on it again, repeating the sequence as quickly as possible. The score was calculated as the sum of one point for each board movement and one point for each body transfer movement on the board. The task consisted of accumulating as many points as possible in 20 s for both trials.

The JS subtest consisted of two-legged lateral jumps performed as quickly as possible in 15 s over a wooden beam (60 cm × 4 cm × 2 cm). After an initial familiarization phase involving five jumps, each participant performed two trials. The task consisted of executing as many jumps as possible to the left and right over the beam. The score was calculated as the sum of jumps completed in both trials.

The HH subtest consisted of one-legged hopping for height over an obstacle of increasing height (i.e., consecutive increments of 5 cm). Each participant started approximately 1.5 m away from a pile of foam pads (each measuring 60 cm × 20 cm × 5 cm). After a short run-up, each participant jumped over the obstacles, landed, and performed another jump on the same foot without letting the other foot touch the ground. The starting height of the obstacle varied according to age (6 years: 5 cm/1 pad; 7–8 years: 15 cm/3 pads; 9–10 years: 25 cm/5 pads; 11–14 years: 35 cm/7 pads). The task was performed with both the right and left foot. When the jump trial at a given height was successfully completed with both feet, a pad was added and the test was repeated at an increased height. The score was calculated as three points for successful trial on the first attempt, two points on the second attempt, and one point on the third attempt. The test continued until the sum of the two most recent jump trials for the same foot was less than five points, at which point the test for that foot was terminated. The maximum score per leg was 39 points, including an initial score of three points for successfully completing the jump trial at the first height. The score was calculated as the sum of the scores for both legs.

In the KTK, MC is expressed using the Motor Quotient (MQ), representing the overall gross motor coordination, calculated by summing the four subtest scores and norm-referenced for age and sex. In detail, the raw scores of the four subtests were summed in a total raw score. Furthermore, the raw scores of the four subtests were normalized using age- and sex-specific normative tables, and their sum yielded the raw motor quotient. Finally, the MQ was computed using standardized conversion tables.

#### 2.3.3 Physical activity level assessment

The level of PA was assessed using the Physical Activity Questionnaire for Children (PAQ-C), a self-reported questionnaire designed to measure the level of PA practiced over the previous seven days in children aged 8 to 14 years (38). The questionnaire consists of a series of closed-ended questions and provides a mean score ranging from 1 to 5, where higher values indicate a higher frequency and intensity of PA.

The questionnaire includes 10 items regarding participation in sports and PAs and is administered for each day of the previous week. Participants were asked to indicate the type of sport and/or PA they engaged during each day of the previous week, both at home and at school, including: (i) whether and many times they engaged in any of the 22 types of sports and PAs listed, or other activities not listed, in the previous seven days; (ii) whether and many times they engaged intensively in PE classes in the previous seven days; (iii) what they typically did during school recess or breaks; (iv) what PA they engaged during lunch breaks in addition to eating; (v) many times they were very physically active on weekday afternoons, evenings, or weekends; (vi) selecting, from five possible options, the statement that best represented the overall amount and type of PA performed; (vii) the frequency of PA performed on each day of the previous week (Monday to Sunday).

Each question was scored and the total score was calculated as the mean of the scores for all questions.

### 2.4 Statistical analysis

Means ± standard deviations (SD) were computed for descriptive statistics of participants' characteristics, MC performances, and PAQ-C level. The Shapiro–Wilk test was used to verify data normality.

A one-way Analysis of Variance (ANOVA) was used to analyse any difference in height, weight, and BMI among the three independent cohorts (i.e., pre-COVID-19, post-COVID-19, and follow-up COVID-19 restrictions).

A three-way Analysis of Variance (ANOVA) was computed to determine the effect of three categorical independent variables, or factors on a single continuous dependent variable (MC performance). In detail, it was used to assess the main effects for each factor (i.e., Time, Age, Sex), three two-way interactions (i.e., Time * Age; Time * Sex; Age * Sex), and one three-way interaction (i.e., Time * Age * Sex) on performance of each KTK subtest (i.e., WB, JS, MS, HH), as well as on performance of global MC (i.e., MQ), and on PAQ-C level.

Partial eta-squared (*η*^2^_p_) was used to assess the effect size, considering *η*^2^_p_ = 0.01 as small, *η*^2^_p_ = 0.06 as medium, and *η*^2^_p_ = 0.14 as large effect (39).

In case of significant factors main effects, or significant interaction effect, Bonferroni *post-hoc* tests were carried out.

All statistical analyses and graphs were performed using Jamovi (The jamovi project (2024). jamovi (Version 2.5) [Computer Software]. Retrieved from https://www.jamovi.org) with the significance level set at *p* < 0.05.

All graphs were carried out using GraphPad Prism 8 (GraphPad Software Inc., San Diego, CA, USA).

## 3 Results

### 3.1 Participants

Overall, 449 school aged children were included from three independent cohorts (i.e., in pre-COVID-19 *n* = 141 of which *n* = 70 females and *n* = 71 males; in post-COVID-19 *n* = 139 of which *n* = 69 females and *n* = 70 males; in follow-up COVID-19 restrictions *n* = 169 of which *n* = 75 females and *n* = 94 males). No significant differences were found in height (*p* = 0.856), weight (*p* = 0.278), and BMI (*p* = 0.074) across the three cohorts. The Shapiro–Wilk test showed that data were normally distributed.

**Figure 1:**
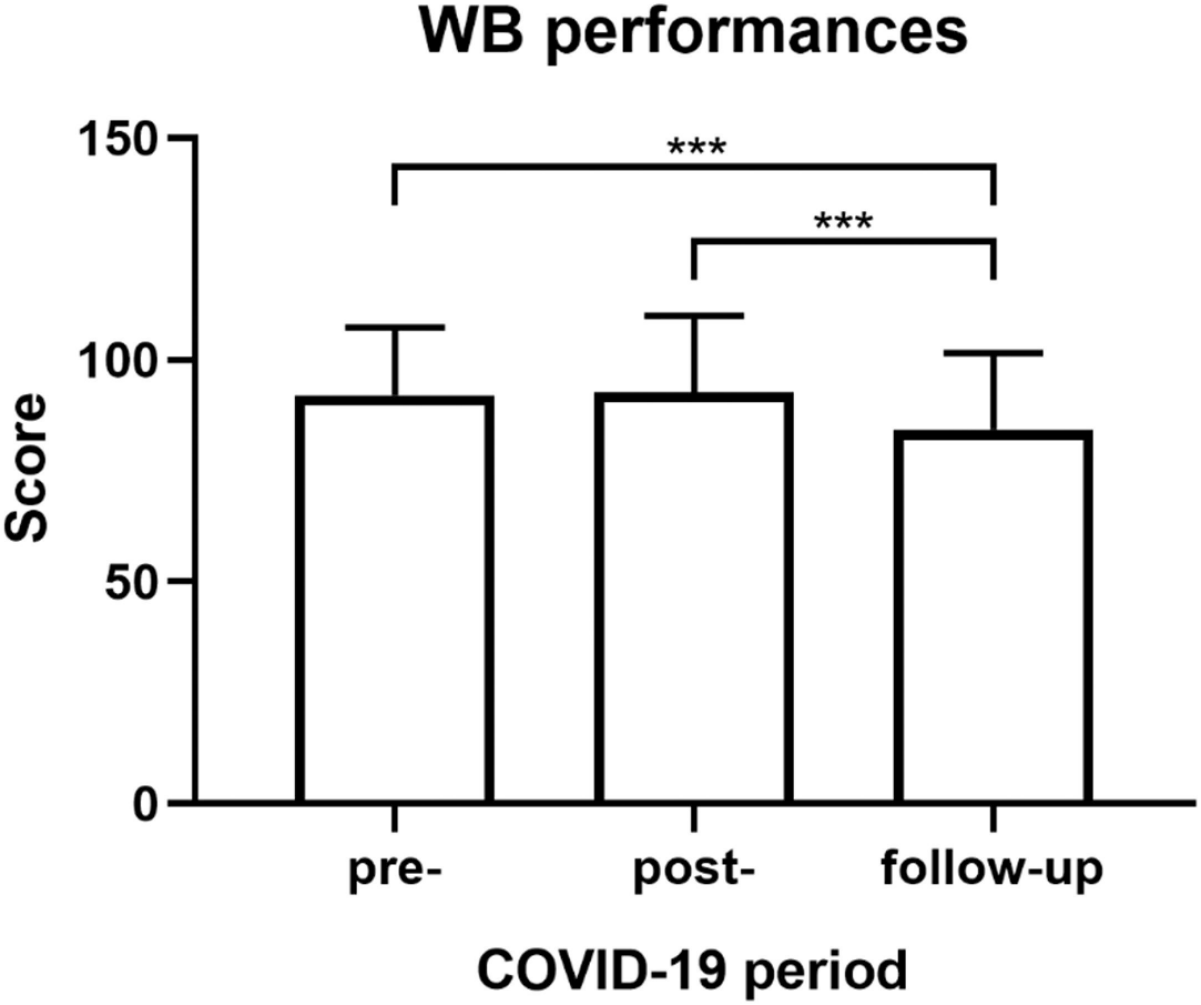
WB performances across the three COVID-19 periods. WB, walking backward; *, *p* < 0.05; **, *p* < 0.01; ***, *p* < 0.001.

**Figure 2:**
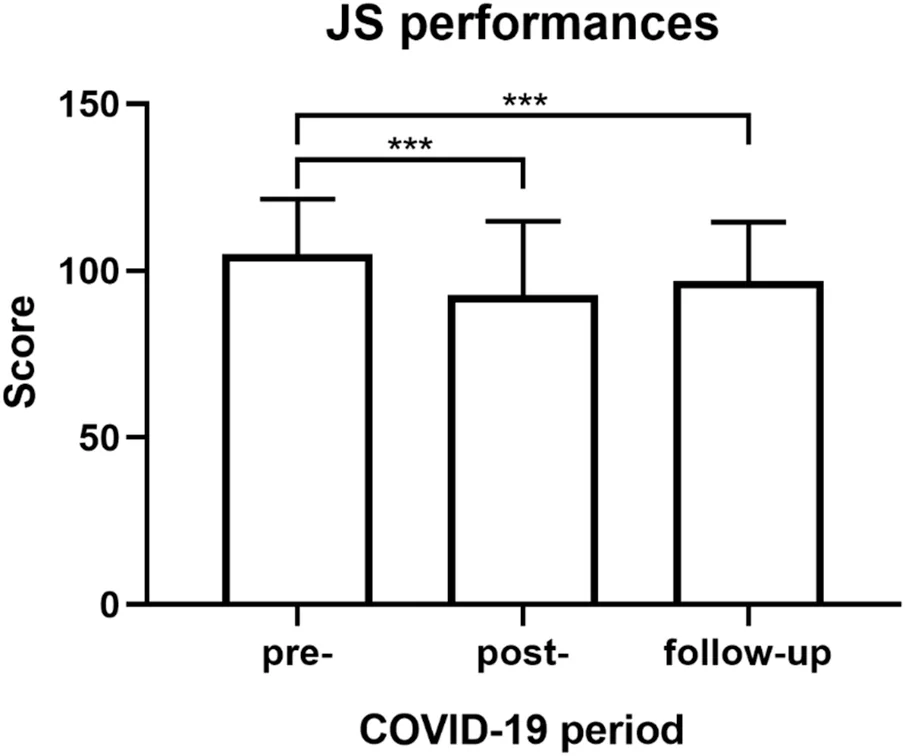
JS performances across the three COVID-19 periods. JS, two-legged jumping sideways; *, *p* < 0.05; **, *p* < 0.01; ***, p < 0.001.

**Figure 3:**
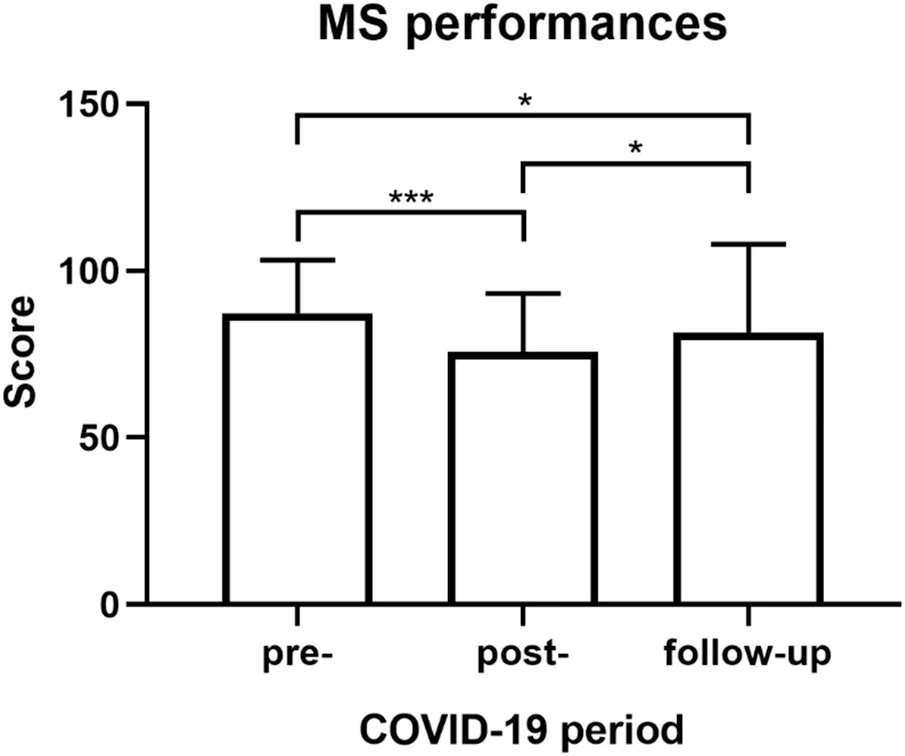
MS performances across the three COVID-19 periods. MS, moving sideways; *, *p* *<* 0.05; **, *p* *<* 0.01; ***, *p* *<* 0.001.

**Figure 4:**
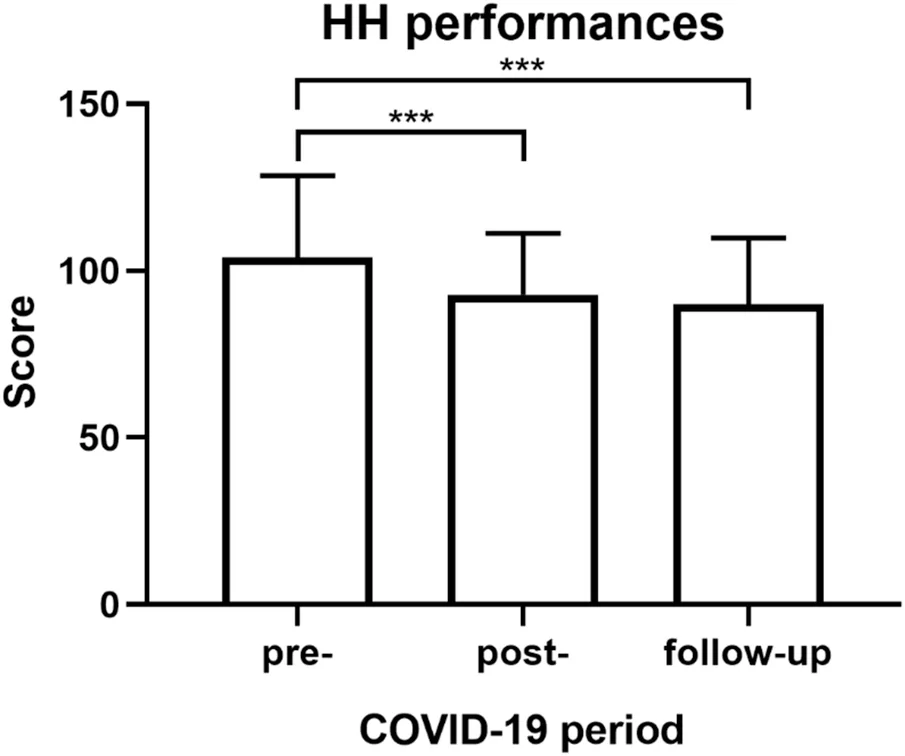
HH performances across the three COVID-19 periods. HH, one-legged hopping for height; *, *p* < 0.05; **, *p* < 0.01; ***, *p* < 0.001.

**Figure 5:**
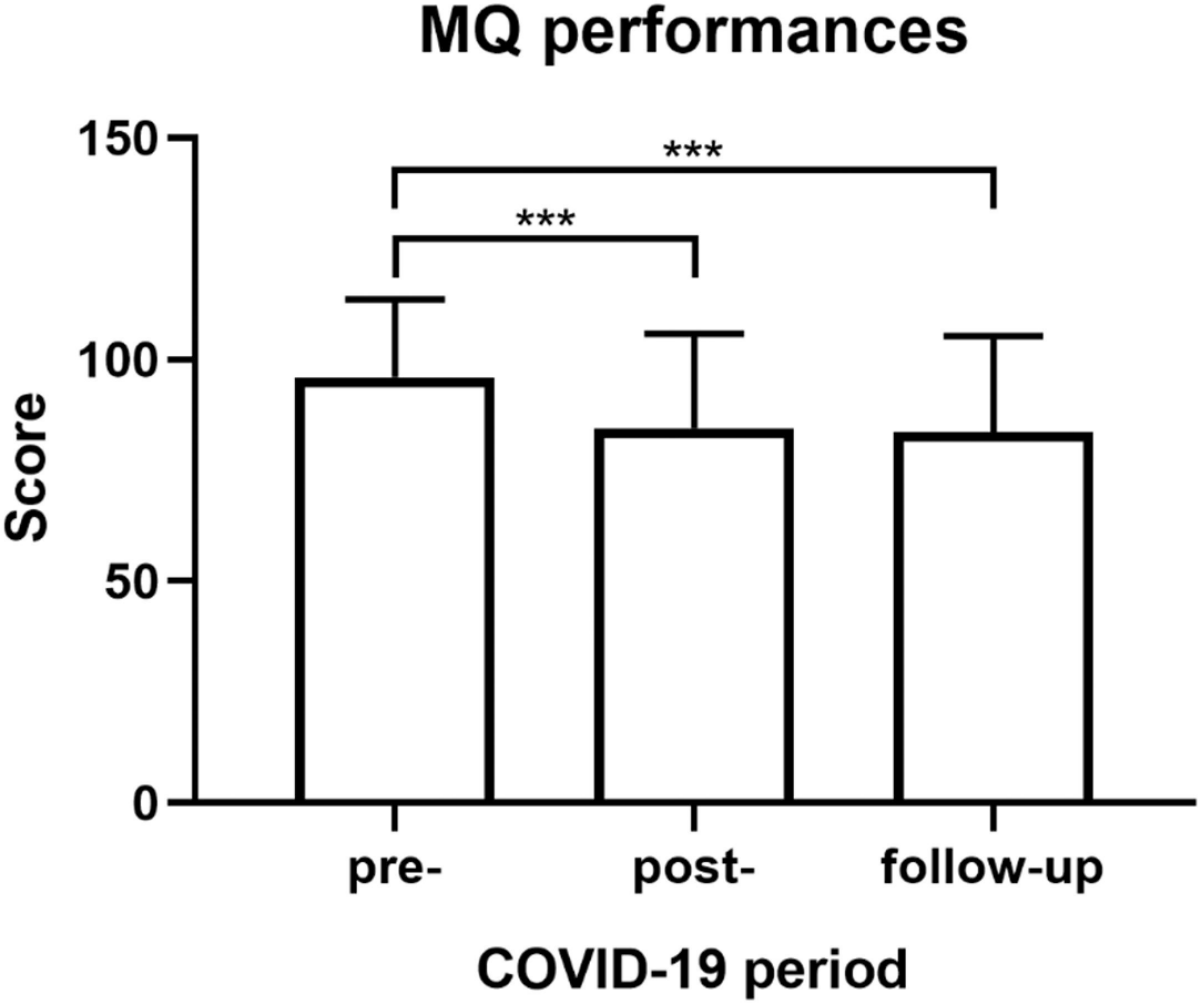
MQ performances across the three COVID-19 periods. MQ, motor quotient; *, *p* *<* 0.05; **, *p* *<* 0.01; ***, *p* *<* 0.001.

**Figure 6:**
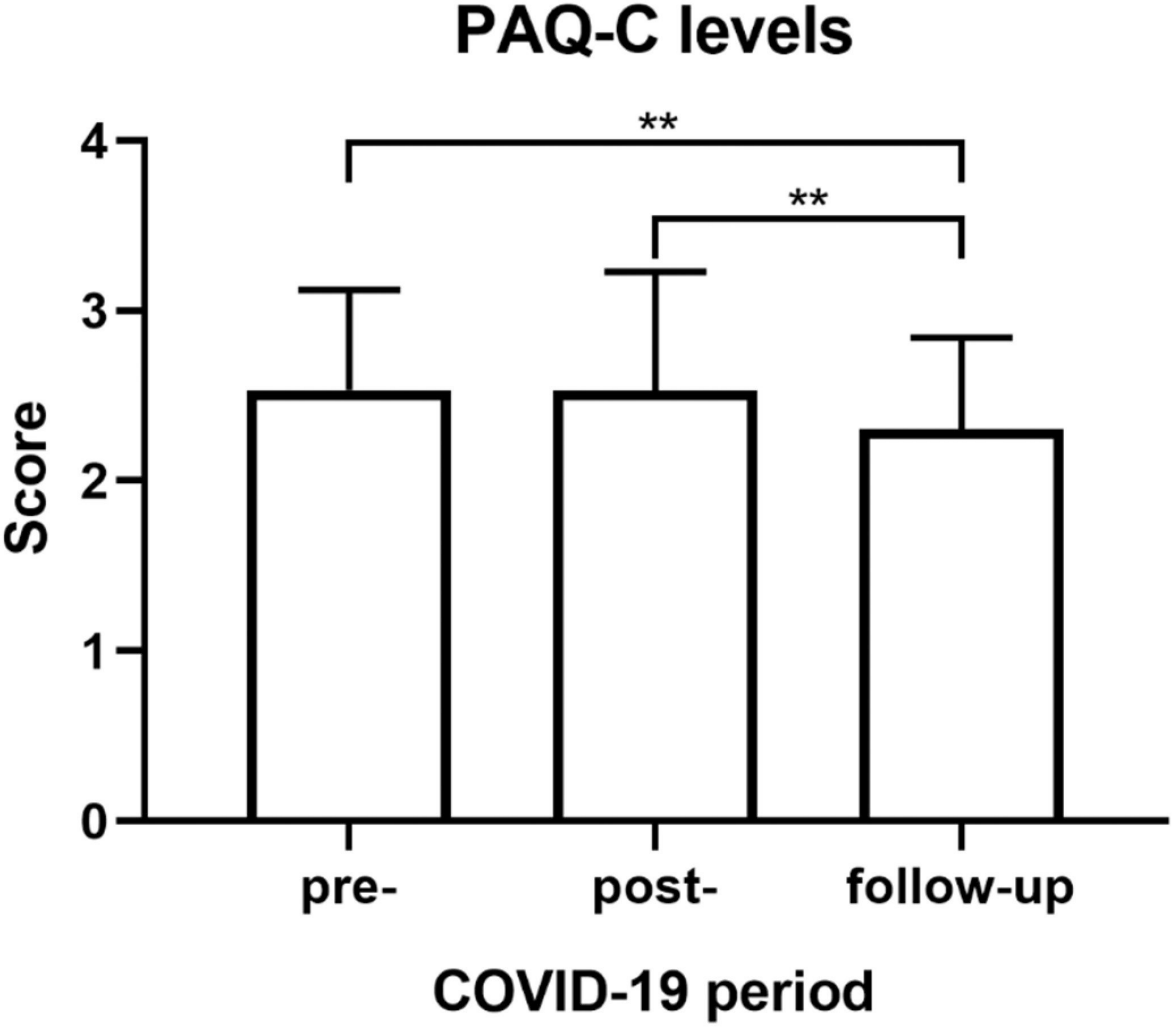
PAQ-C levels across the three COVID-19 periods. PAQ-C, physical activity questionnaire for children; *, *p* < 0.05; **, *p* < 0.01; ***, *p* < 0.001.

Table 1 provides an overview of the sample characteristics (age, sex, height, weight, and BMI) according to the three COVID-19 periods. Descriptive statistics of participants' MC performances and PAQ-C level pre-, post-, and follow-up COVID-19 restrictions, detailed for age and sex, are reported in Table 2. Figures 1–5 shows the level of each subtest performances and of the MQ of the KTK, regardless of participants' age and sex, according to the three COVID-19 periods. Figure 6 shows the level of PA of the participants, regardless of age and sex.

**Table 1:** Participants' characteristics across the three COVID-19 periods.

COVID-19 period	Age (years)	Sex	*N*	Height (m)	Weight (kg)	BMI (kg/m^2^)
Pre-	8	F	15	1.31 ± 0.06	31.17 ± 8.04	18.09 ± 4.12
		M	22	1.32 ± 0.05	30.46 ± 5.00	17.35 ± 2.16
	9	F	20	1.40 ± 0.06	37.88 ± 10.27	19.21 ± 4.55
		M	26	1.41 ± 0.08	36.81 ± 10.17	18.12 ± 3.29
	10	F	35	1.45 ± 0.09	38.89 ± 12.00	18.26 ± 3.87
		M	23	1.46 ± 0.09	39.82 ± 8.87	18.51 ± 2.62
TOT	8–10	M–F	141	1.40 ± 0.09	36.38 ± 10.13	18.25 ± 3.49
Post-	8	F	22	1.31 ± 0.04	28.11 ± 6.14	16.25 ± 2.75
		M	22	1.33 ± 0.07	31.59 ± 6.08	17.92 ± 2.67
	9	F	23	1.41 ± 0.07	33.96 ± 8.30	16.99 ± 2.97
		M	21	1.40 ± 0.06	33.80 ± 7.20	17.20 ± 2.75
	10	F	24	1.45 ± 0.09	38.78 ± 10.30	18.10 ± 2.75
		M	27	1.49 ± 0.06	39.74 ± 8.15	17.88 ± 2.80
TOT	8–10	M–F	139	1.40 ± 0.09	34.59 ± 8.75	17.42 ± 2.81
follow-up	8	F	22	1.30 ± 0.05	27.21 ± 5.11	16.11 ± 2.78
		M	31	1.34 ± 0.07	29.34 ± 6.01	16.38 ± 2.79
	9	F	24	1.39 ± 0.07	34.03 ± 8.39	17.43 ± 3.31
		M	30	1.40 ± 0.06	36.98 ± 9.02	18.81 ± 3.29
	10	F	29	1.47 ± 0.09	44.47 ± 13.56	20.17 ± 4.50
		M	33	1.45 ± 0.08	39.28 ± 9.39	18.38 ± 2.89
TOT	8–10	M–F	169	1.40 ± 0.09	35.62 ± 10.68	17.97 ± 3.56

*N*, number; m, meter; kg, kilograms.

**Table 2:** Participants' MC performances and PAQ-C level across the three COVID-19 periods, detailed for age and sex.

COVID-19 period	Variable	Age (years)	F (Mean ± SD)	M (Mean ± SD)
Pre-	WB	8	94.00 ± 16.29	91.14 ± 15.49
Post-	WB	8	99.91 ± 17.16	92.73 ± 14.55
Follow-up	WB	8	83.50 ± 13.53	83.23 ± 13.50
Pre-	WB	9	92.85 ± 13.64	90.35 ± 15.04
Post-	WB	9	95.78 ± 18.71	95.33 ± 15.01
Follow-up	WB	9	94.25 ± 13.34	80.41 ± 19.80
Pre-	WB	10	91.71 ± 18.47	93.74 ± 10.35
Post-	WB	10	89.25 ± 19.03	85.19 ± 16.03
Follow-up	WB	10	83.89 ± 22.04	82.58 ± 16.52
Pre-	JS	8	98.33 ± 16.56	108.77 ± 13.54
Post-	JS	8	97.55 ± 21.41	109.00 ± 15.83
Follow-up	JS	8	91.73 ± 16.27	107.93 ± 17.79
Pre-	JS	9	96.10 ± 17.36	116.08 ± 11.99
Post-	JS	9	82.04 ± 21.92	104.24 ± 15.52
Follow-up	JS	9	89.46 ± 15.48	98.35 ± 14.68
Pre-	JS	10	98.69 ± 17.68	109.87 ± 11.46
Post-	JS	10	72.79 ± 15.85	92.96 ± 20.01
Follow-up	JS	10	89.00 ± 18.76	101.32 ± 15.60
Pre-	MS	8	84.73 ± 19.27	85.96 ± 17.19
Post-	MS	8	74.36 ± 20.53	80.14 ± 16.00
Follow-up	MS	8	76.23 ± 34.09	71.83 ± 38.28
Pre-	MS	9	84.35 ± 13.92	90.50 ± 14.72
Post-	MS	9	72.74 ± 17.87	77.24 ± 16.48
Follow-up	MS	9	91.63 ± 16.62	77.66 ± 22.40
Pre-	MS	10	87.97 ± 15.85	88.13 ± 16.43
Post-	MS	10	72.88 ± 16.76	76.07 ± 18.71
Follow-up	MS	10	83.36 ± 19.44	88.68 ± 17.12
Pre-	HH	8	105.00 ± 29.40	110.27 ± 25.97
Post-	HH	8	97.05 ± 19.67	97.41 ± 15.17
Follow-up	HH	8	83.82 ± 15.28	94.27 ± 15.95
Pre-	HH	9	100.05 ± 25.53	102.50 ± 20.94
Post-	HH	9	90.17 ± 16.59	95.52 ± 14.77
Follow-up	HH	9	89.29 ± 16.86	90.86 ± 19.68
Pre-	HH	10	100.09 ± 27.40	107.83 ± 18.02
Post-	HH	10	82.79 ± 19.91	94.52 ± 20.24
Follow-up	HH	10	85.54 ± 27.33	93.87 ± 20.10
Pre-	MQ	8	94.00 ± 19.57	98.59 ± 17.79
Post-	MQ	8	89.77 ± 20.94	93.18 ± 15.38
Pollow-up	MQ	8	79.00 ± 17.02	85.00 ± 21.01
Pre-	MQ	9	91.25 ± 15.80	99.54 ± 15.74
Post-	MQ	9	80.74 ± 21.50	90.81 ± 17.47
Pollow-up	MQ	9	88.46 ± 15.00	82.76 ± 21.15
Pre-	MQ	10	92.89 ± 20.24	99.78 ± 14.25
Post-	MQ	10	71.83 ± 23.48	83.30 ± 21.00
Pollow-up	MQ	10	78.46 ± 29.08	87.87 ± 21.97
Pre-	PAQ-C	8	2.23 ± 0.79	2.57 ± 0.57
Post-	PAQ-C	8	2.49 ± 0.62	2.63 ± 0.79
Pollow-up	PAQ-C	8	2.26 ± 0.44	2.38 ± 0.72
Pre-	PAQ-C	9	2.40 ± 0.62	2.55 ± 0.50
Post-	PAQ-C	9	2.45 ± 0.64	2.61 ± 0.56
Pollow-up	PAQ-C	9	2.38 ± 0.50	2.22 ± 0.40
Pre-	PAQ-C	10	2.61 ± 0.61	2.68 ± 0.44
Post-	PAQ-C	10	2.17 ± 0.59	2.83 ± 0.80
Follow-up	PAQ-C	10	2.29 ± 0.60	2.31 ± 0.51

WB, walking backward; MS, moving sideways; JS, two-legged jumping sideways; HH, one-legged hopping for height; MQ, motor quotient; PAQ-C, physical activity questionnaire for children.

### 3.2 Motor coordination

As concerns the KTK subtests performances, the three-way ANOVA showed a significant difference across the three COVID-19 periods in the WB subtest scores [F_(2,426)_ = 12.106, *p* < 0.001, *η*^2^ = 0.054] and in the Sex factor [F_(1,426)_ = 4.534, *p* = 0.034, *η*^2^ = 0.011]. Bonferroni *post-hoc* test showed a significant effect from pre- to follow-up (*p* < 0.001) and from post to follow-up (*p* < 0.001) with females reporting higher WB performances compared to males (*p* = 0.034), as reported in Table 3.

**Table 3:** Three-way ANOVA and *post-hoc* comparisons for the WB subtest.

Three-way ANOVA			
Factor	F	*p*	*η* ^2^_p_
Time	12.106	< 0.001***	0.054
Age	2.268	0.105	0.011
Sex	4.534	0.034*	0.011
Time * age	1.766	0.135	0.016
Time * sex	0.558	0.573	0.003
Age * sex	0.706	0.494	0.003
Time * age * sex	1.358	0.248	0.013
Post-hoc comparisons (time factor)			
	Mean difference	*p*	Cohen's *d*
Pre vs. post	−0.734	0.714	−0.045
Pre vs. follow-up	7.653	< 0.001***	0.465
Post vs. follow-up	8.386	< 0.001***	0.510
Post-hoc comparisons (sex factor)			
	Mean difference	*p*	Cohen's *d*
F vs. M	3.384	0.034*	0.206

WB, walking backward; *, *p* < 0.05; ***, *p* < 0.001.

As for the JS subtest scores, we detected a significant main effect for all factors (i.e., Time, Age, Sex) and for one two-way interaction (i.e., Time * Age) with the same level of significance (*p* < 0.001). Bonferroni *post-hoc* test revealed a significant difference between pre- and post- (*p* < 0.001) and between pre- and follow-up (*p* < 0.001) for the Time factor, between 8 and 9 years old (*p* = 0.029) and between 8 and 10 years old (*p* < 0.001) for the Age factor, as well as between the Sex factor (*p* < 0.001). Table 4 also shows the main significant differences found in the interaction Time * Age (i.e., the interaction between each age group across the different COVID-19 periods) for the JS subtest.

**Table 4:** Three-way ANOVA and *post-hoc* comparisons for the JS subtest.

Three-way ANOVA			
Factor	F	*p*	*η* ^2^_p_
Time	17.024	<0.001***	0.074
Age	8.400	<0.001***	0.038
Sex	82.788	<0.001***	0.163
Time * age	5.369	<0.001***	0.048
Time * sex	1.039	0.355	0.005
Age * sex	0.562	0.571	0.003
Time * age * sex	1.303	0.268	0.012
Post-hoc comparisons (time factor)			
	Mean difference	*p*	Cohen's *d*
Pre vs. post	11.543	<0.001***	0.688
Pre vs. follow-up	8.342	<0.001***	0.497
Post vs. follow-up	−3.201	0.101	−0.191
Post-hoc comparisons (age factor)			
	mean difference	*p*	Cohen's *d*
8 vs. 9	4.508	0.029*	0.269
8 vs. 10	8.113	< 0.001***	0.483
9 vs. 10	3.605	0.062	0.215
Post-hoc comparisons (sex factor)			
	Mean difference	*p*	Cohen's *d*
F vs. M	−14.760	< 0.001***	−0.879
Post-hoc comparisons (time * age interaction)			
	Mean difference	*p*	Cohen's *d*
Pre * 8 vs. post*8	0.280	0.941	0.017
Pre * 8 vs. follow-up * 8	3.723	0.311	0.222
Post * 8 vs. follow-up * 8	3.442	0.320	0.205
Pre * 9 vs. post * 9	12.948	<0.001***	0.771
Pre * 9 vs. follow-up * 9	12.187	<0.001***	0.726
Post * 9 vs. follow-up * 9	−0.761	0.825	−0.045
Pre * 10 vs. post * 10	21.400	<0.001***	1.275
Pre * 10 vs. follow-up * 10	9.116	0.004**	0.543
Post * 10 vs. follow-up * 10	−12.284	<0.001***	−0.732

JS, two-legged jumping sideways; *, *p* < 0.05; **, *p* < 0.01; ***, *p* < 0.001.

Table 5 reports statistical analyses for the MS subtest performances. In detail, a significant difference across the three COVID-19 periods was found [F_(2,426)_ = 10.148, *p* < 0.001, *η*^2^ = 0.045] with a significant change from pre- to post-COVID-19 (*p* < 0.001), from pre- to follow-up (*p* = 0.028), and from post- to follow-up (*p* = 0.013). No other main effects factors nor interactions effects were detected (*p* > 0.05).

**Table 5:** Three-way ANOVA and *post-hoc* comparisons for the MS subtest.

Three-way ANOVA			
Factor	F	*p*	*η* ^2^_p_
Time	10.148	< 0.001***	0.045
Age	1.487	0.227	0.007
Sex	0.194	0.660	0.000
Time * age	1.899	0.110	0.018
Time * sex	1.893	0.152	0.009
Age * sex	0.353	0.703	0.002
Time * age * sex	1.422	0.226	0.013
Post-hoc comparisons (time factor)			
	Mean difference	*p*	Cohen's *d*
Pre vs. post	11.369	< 0.001***	0.548
Pre vs. follow-up	5.377	0.028*	0.259
Post vs. follow-up	−5.992	0.013*	−0.289

MS, moving sideways; *, *p* < 0.05; ***, *p* < 0.001.

In the HH subtest scores, the main effect of the Time and of the Sex were significant [F_(2,426)_ = 18.967, *p* < 0.001, *η*^2^ = 0.082 and F_(1,426)_ = 8.528, *p* = 0.004, *η*^2^ = 0.020, respectively]. The *post-hoc* analysis indicated that the HH performances during pre-COVID-19 were significantly higher than post-COVID-19 (*p* < 0.001) as well as compared to the follow-up (*p* < 0.001). Moreover, females had lower HH performances compared to males (*p* = 0.004). Table 6 shows HH subtest analyses.

**Table 6:** Three-way ANOVA and *post-hoc* comparisons for the HH subtest.

Three-way ANOVA			
Factor	F	*p*	*η* ^2^_p_
Time	18.967	< 0.001***	0.082
Age	1.342	0.262	0.006
Sex	8.528	0.004**	0.020
Time * age	0.891	0.469	0.008
Time * sex	0.056	0.945	0.000
Age * sex	0.844	0.431	0.004
Time * age * sex	0.435	0.783	0.004
Post-hoc comparisons (time factor)			
	Mean difference	*p*	Cohen's *d*
Pre vs. post	11.379	< 0.001***	0.543
Pre vs. follow-up	14.682	< 0.001***	0.700
Post vs. follow-up	3.303	0.175	0.157
Post-hoc comparisons (sex factor)			
	Mean difference	*p*	Cohen's *d*
F vs. M	−5.918	0.004**	−0.282

HH, one-legged hopping for height; **, *p* < 0.01; ***, *p* < 0.001.

Regarding the MQ scores, significant Time effect [F_(2,426)_ = 16.161, *p* < 0.001, *η*^2^ = 0.071] and Sex effect [F_(1,426)_ = 9.825, *p* = 0.002, *η*^2^ = 0.023] were noted. The *post-hoc* analysis showed a significant decrease from pre-COVID-19 to post-COVID-19 (*p* < 0.001) as well as from pre-COVID-19 to follow-up (*p* < 0.001). Furthermore, females reported lower MQ performances compared to males (*p* = 0.002). All details concerning the MQ analyses are reported in Table 7.

**Table 7:** Three-way ANOVA and *post-hoc* comparisons for the MQ scores.

Three-way ANOVA			
Factor	F	*p*	*η* ^2^_p_
Time	16.161	< 0.001***	0.071
Age	1.847	0.159	0.009
Sex	9.825	0.002**	0.023
Time * age	2.318	0.056	0.021
Time * sex	0.632	0.532	0.003
Age * sex	0.749	0.474	0.004
Time * age * sex	0.966	0.426	0.009
Post-hoc comparisons (time factor)			
	Mean difference	*p*	Cohen's *d*
Pre vs. post	11.069	<0.001***	0.554
Pre vs. follow-up	12.416	<0.001***	0.622
Post vs. follow-up	1.347	0.561	0.067
Post-hoc comparisons (sex factor)			
	Mean difference	*p*	Cohen's *d*
F vs. M	−6.047	0.002**	−0.303

MQ, motor quotient; **, *p* < 0.01; ***, *p* < 0.001.

### 3.3 Physical activity level

The levels of PA, assessed through the PAQ-C, showed significant Time effect [F_(2,410)_ = 6.140, *p* = 0.002, *η*^2^ = 0.029] and Sex effect [F_(1,410)_ = 7.894, *p* = 0.005, *η*^2^ = 0.019]. *Post-hoc* comparisons for the Time factor revealed a significant decrease of the level of PA practice from pre- to follow-up (*p* = 0.006) and from post- to follow-up (*p* = 0.002). As for the Sex factor, females had lower PA levels compared to males (*p* = 0.005). Table 8 reported the statistical analysis for the PAQ-C scores.

**Table 8:** Three-way ANOVA and *post-hoc* comparisons for the PAQ-C scores.

Three-way ANOVA			
Factor	F	*p*	*η* ^2^_p_
Time	6.140	0.002**	0.029
Age	0.353	0.703	0.002
Sex	7.984	0.005**	0.019
Time * age	0.919	0.453	0.009
Time * sex	2.760	0.064	0.013
Age * sex	1.027	0.359	0.005
Time * age * sex	1.505	0.200	0.014
Post-hoc comparisons (time factor)			
	Mean difference	*p*	Cohen's *d*
Pre vs. post	−0.021	0.776	−0.035
Pre vs. follow-up	0.202	0.006**	0.336
Post vs. follow-up	0.223	0.002**	0.371
Post-hoc comparisons (sex factor)			
	Mean difference	*p*	Cohen's *d*
F vs. M	−0.168	0.005**	−0.279

PAQ-C, physical activity questionnaire for children; **, *p* < 0.01.

## 4 Discussion

The aim of this study was to explore any changes in MC levels in Italian primary school children immediately before the COVID-19 pandemic, immediately after the COVID-19 restrictions, and after 3 years from the end of the state of emergency for COVID-19 pandemic to investigate how the long-term effects of COVID-19 restrictions were associated with MC.

This investigation could be possible by recruiting children between 8 and 10 years of three independent cohorts (i.e., in 2019, in 2022, and in 2025, for analysing data pre-pandemic, post-pandemic, and follow-up pandemic, respectively). When comparing the pre-pandemic cohort with both the post-pandemic cohort and the follow-up cohort, we found higher MC performances. Hence, the COVID-19 restrictions were associated with decreased levels of MC in Italian school-aged children between 8 and 10 years.

These findings are in line with most of the existing literature. Indeed, previous studies have shown that during and after COVID-19 pandemic the levels of MC in children decreased compared to before the pandemic (21, 40, 41). This could be mainly explained by the forced reduction in PA practice. As a matter of fact, physical inactivity in children is considered an epidemic and has previously been identified as one of the consequences related to the COVID-19 pandemic (42, 43). Of interest, a systematic review and multilevel meta-analysis of 173 studies including 320,636 participants was conducted with the aim of quantifying changes in PA and identifying variables potentially predicting PA reductions during COVID-19 restrictions (44). The group of researchers found that the restrictions associated with the COVID-19 pandemic have led to a moderate reduction in PA levels and a large increase in sedentary behaviours.

In a longitudinal study in Portuguese children, authors detected lower global motor skills performance in the post-COVID-19 compared to pre-COVID-19 assessment and below average for their age, highlighting the negative impact that pandemic lockdown may have had on children's motor development probably due to changes in daily routines, behaviours, stimuli, and activities that children have experienced during the lockdown (45). In line with our main interpretation regarding the forced reduction of PA during COVID-19 restrictions, a four-year follow-up study in Portuguese children aimed at examining the possible long-term effects on children's MC, by assessing a sample of sixty-seven children before, after, and four years apart from COVID-19 lockdown, showed that the MC scores after COVID-19 lockdown was lower on the subscales and on the total MC of the test used for all children (46). After the COVID-19 lockdown, MC were lower in the subscales scores and in the total score of the test used for all children.

In particular, for the WB performances, a study by Martínez-Córcoles et al. detected that the decrease in PA, associated with the pandemic restrictions, affected body balance in children and this was more evident in those with higher levels of PA compared to children who had lower levels of PA (47). The finding that females performed better than males on the backward walking balance subtest is in line with the results of previous research reporting gender differences in the development of postural control (26, 48–50), due to a faster maturation of the vestibular system and of sensory integration ability (51–53).

The reduction in the performance of specific subtests, such as JS and HH, in which the use of strength in the lower limbs is required could be interpreted by the fact that upper and lower limb strength is greatly developed through structured practice in PE and organized sports, activities unavailable during the pandemic (21, 40). Regarding sex and age differences, the lower performance observed in females and older children is also supported by previous research reporting that females and older age groups showed lower levels of PA during the pandemic and this could be due to the greater dependence on routine and the lack of alternative programs for organized activities (43).

The trend also found for the MS scores reflects previous findings as reported by Pombo et al. in which the ability to move sideways for 20 s using two wooden platforms was found to have a significant negative impact following the pandemic (54). Authors argued these results by considering as main factor the time spent in PA and the increase in sedentary behaviours such as screen-based activities.

In the theoretical framework of Stodden et al. it has been developed that PA promotes MC through a variety of physical and structured activities (e.g., sports activities) (55). In fact, higher levels of MC determine a greater practice of PA and, reciprocally, a greater practice of PA leads to a greater MC. And, if the practice of organized exercise is partially or totally excluded from children's habits, MC may not be developed in an appropriate manner.

Nonetheless, caution is warranted regarding the possible causal effects of COVID-19 restrictions and related practice of PA on MC. Indeed, the multitude of factors related to COVID-19 restrictions that may have led to a decrease in children's MC levels may not actually be directly dependent on children's PA levels (e.g., home environment, or screen time). Furthermore, the variables that interact in the development of children's MC are multiple and complex and were not considered in this study (e.g., maturation, or organized sport participation). Therefore, caution should be used in drawing general conclusions.

In fact, in contrast with our results, a study by den Uil et al. (2023), aimed at comparing motor skill development over one year among four different cohorts of Netherlands primary school-age children (of which two experienced no lockdowns and two experienced one or two lockdowns for a total of 992 children between 6 and 7 years), showed that COVID-19 lockdowns did not negatively affect motor skill development (56).

In conclusion, in this study we explored how COVID-19 pandemic was associated with changes in MC levels in Italian school-aged children using a large, representative sample. The results underline the negative association between pandemic restrictions and MC. As demonstrated by several authors, the decrease in children's MC levels was probably influenced negatively by the suspension from PAs and sports activities (54). Considering that MC in children positively influences the practice of PA and sports throughout life (7, 57), and taking into account the long-term results we found, it should be developed solutions to raise awareness among the general population and increase, from the very beginning, the amount of physical and sporting activities in children. Furthermore, these findings should prompt reflection on possible interventions to be adopted during periods when, for some reason not necessarily related to restrictions on public life, PAs cannot be practiced regularly, especially in children where motor development may be affected.

The main limitation of this study is that, although we compared homogeneous groups, belonging to the same geographical area and with the same socioeconomic background, the current exploration compared independent cohorts and did not longitudinally assess the same sample. Among the limitations, it should be reported that the use of a self-reported questionnaire to measure the level of PA practiced over the previous seven days could introduce a risk of recall bias and reporting bias (i.e., social desirability bias).

## Data Availability

The raw data supporting the conclusions of this article will be made available by the authors, without undue reservation.
